# Building Teachers' Resilience: Practical Applications for Teacher Education of China

**DOI:** 10.3389/fpsyg.2021.738606

**Published:** 2021-08-12

**Authors:** Yi Wang

**Affiliations:** International Education College, Henan University, Kaifeng, China

**Keywords:** teacher resilience, teaching, teacher education, Chinese teacher education, practical applications

## Abstract

Teacher resilience has a crucial role to play in teaching and teacher education all around the world. However, few practical attempts have been made to systematically improve and (re)build this characteristic in teachers. Against this backdrop, this article draws on a universal model to offer practical implications of building resilience in the teacher education of China which is largely oriented toward pedagogical and economic concerns rather than the socio-emotional aspects of teaching. More particularly, it explains the history of China's teacher education, the conceptualizations and significance of teacher resilience, and a systematic model to integrate resilience into teacher education. Finally, some practical implications and future directions are provided for avid scholars.

## Introduction

Undoubtedly, teaching is one of the most challenging and complicated professions in the world owing to its intellectual, emotional, and service-providing nature (Mercer, 2020; Sikma, 2021). Teachers as the “pillars of societies,” need to know “what to teach,” “how to teach,” and “cope with educational adversities and challenges” at the same time (Pishghadam et al., 2021; Sikma, 2021). This justifies the necessity of a shift of attention from students' psychology to teachers' psychology and emotion which came into vogue with the arrival of positive psychology (PP) that capitalized on “how people flourish” instead of lingering on “negative stressors” (MacIntyre and Mercer, 2014). As teachers carry their own feelings, emotions, and values to the class, caring for their mental well-being and inner states is of utmost significance in all educational contexts (Dewaele and Li, 2020; Zhang and Zhang, 2020; Greenier et al., 2021; Wang and Derakhshan, 2021a). However, in reality, there emerge numerous challenges and setbacks in teaching which causes teachers' attrition, demotivation, stress, and burnout especially during the first 5 years of teaching which is known as “the vulnerability period” during which 40–50% of teachers quit their job (Gallant and Riley, 2014). This may happen due to a high workload, limited support, fear of challenges, lack of time management, and lack of knowledge about how to control students' behaviors and satisfy their needs (Kelly et al., 2018).

The reason beneath these problems in many countries including China is the inadequacy of teacher education programs which do not prepare teachers for the reality of their job, its tensions and challenges, and ways to go about such adversities and stay strong. They solely dwell upon developing teachers' pedagogical skills and students' test performance without working on the social and emotional aspects of teaching. That is why, a professional teacher who is technically expert in his/her subject is not able to cope with the emotional stressors of his/her profession efficiently, and hence attrition and burnout occur. Consequently, teacher education programs must take a different approach moving from “negative stressors” that make problems for teachers to “positive factors and emotions” which urge teachers to remain in their profession despite its setbacks. One of the most important constructs that boomed in PP trend is teacher resilience which is defined as is a multifaceted, dynamic process comprised of the interaction of personal and contextual resources that permits teachers to bounce back and forth from negative stressors and traumatic events of the field (Li and Yang, 2016; Mansfield et al., 2016). Resilience generates different positive outcomes for teacher education at the macro level and teachers and students at the micro-level. More specifically, it minimizes teachers' stress and burnout, improves their commitment, job satisfaction, well-being, instructional quality, work enjoyment, motivation, professional identity, retention, agency, self-efficacy, and so forth (Brunetti, 2006; Doney, 2013; Richards et al., 2016). Correspondingly, teacher resilience affects students' engagement, motivation, and academic achievement, too (Li et al., 2019a).

This growing body of research signifies the need for integrating teacher resilience into educational systems and teacher education programs worldwide to prepare teachers for coping with the realities of their work. However, few countries like Australia, the United States, Spain, and the Netherlands have taken operational steps to apply a systematic approach to build resilience in their pre/in-service teachers using a groundbreaking model proposed by Mansfield et al. (2016). Nevertheless, in China, with 15 million teachers and 230 million students, the quality of teaching is constantly declining especially in remote districts due to teacher attrition and burnout (Li et al., 2019b). In a context with such regional disparities, teacher education programs are facing a formidable challenge to prepare a resilient teaching force that is both pedagogically and socio-emotionally tough when facing the adversities inherent in teaching a large group of students. Now that a systematic framework that has provided an infusion of insights into teacher education and teaching is available, (re)building teacher resilience in China is by no means a herculean task. In line with this, the present study aimed to explore the practical applications of teachers' resiliency development in teacher education of China.

## Background

### Teacher Education of China

The history of teacher education in China dates back to 1897 when Nanyang College, in Shanghai, began to develop professional teachers (Li, 2016). Then the system went through different reformations which made its developmental trajectory really long. The first movement in teacher education of China was grounded in the Confucian tradition which regarded teachers as the foundation of all education. Unlike Western education which was religion-based, China's education in this period was policy-oriented and societal development was the priority. Next, new educational legislations were laid which divided teacher education into two independent levels for elementary teachers and secondary teachers which offered new visions for teaching in China. Under this rule, teacher education's responsibility was entrusted to independent normal schools and universities. Then with the outbreak of wars, China changed dramatically and to compete against Western powers, the Soviet model of teacher education was implemented for two decades emphasizing the independent teacher education systems by normal schools and universities. Again, at this time, teacher education was mixed with political issues and its main functions were neglected by the Communist government. After that, in the 1980s, to modernize the country, China modified its teacher education and designed compulsory programs for elementary and secondary teachers lasting 3 and 4 years, respectively. Since the 1990s, the rapid growth of globalization and marketization made the Soviet model of no use in this era and China required professionalized and high-quality teachers at all levels. Hence, Chinese policy-makers decided to separate policy and economy from educational decisions which led to a revision and readjustment of the structure and content of the teacher education system. Moreover, knowledgeable and professional teachers, diverse professional development programs, and socio-economically improved teachers were the core purpose of the Chinese teacher education system (see Li, 2016). Although this system has gained many achievements, it still needs to be revised to:

▪ Be driven by teachers' needs rather than political and economic needs.
▪ Improve the quality and equity of education and teacher education.
▪ Take bottom-up steps to care for pedagogical and socio-emotional aspects of teaching and learning (Hu and Verdugo, 2015).

### The Conceptualizations of Resilience

As pinpointed by Beltman (2021), four conceptualizations exist for the concept of resilience. The first conceptualization is *person-focused* and considers resilience as an individual trait manifested during traumatic moments. According to this perspective, a resilient person is one who is able to bounce back in the face of adversity (Doney, 2013). The second conceptualization is *process-focus* or *person-context perspective* which considers resilience as the result of person-context interaction. It defines resilience as a process in which a person, actively, utilizes appropriate strategies to maintain their commitment and well-being in the face of challenges. *Context-focused conceptualization* of resilience argues that aside from individual capacities and strategies, the given context is also paramount. In this perspective, resilience is the ability to adapt to a tense context and maintain one's ability in a challenging socio-cultural context (Johnson et al., 2014). The final conceptualization is *system-focused* which regards resilience as a process with many systems both internal and external to the person which dynamically interact with one another.

### The Significance of Teacher Resilience

Teacher resilience or the ability to stand against the natural stressors and setbacks in teaching as a tough profession is of utmost importance in all educational arenas in that it can generate numerous positive outcomes. More specifically, resiliency produces job satisfaction, responsiveness, effectiveness, self-efficacy, sense of pride, sense of agency, interpersonal relationships, competency, autonomy, optimism, positive interpersonal emotions, empathy, and emotionally intelligent teachers (Tait, 2008; Taylor, 2013; Xie and Derakhshan, 2021). Hence, developing this construct in teachers through rich teacher education programs is a must in academic contexts as teachers are the frontline soldiers in fighting against adversities whose emotional states and readiness makes a great change in educational outcomes worldwide (Derakhshan et al., 2020; Wang and Derakhshan, 2021b).

### A Comprehensive Model of Teacher Resilience

**Figure 1:**
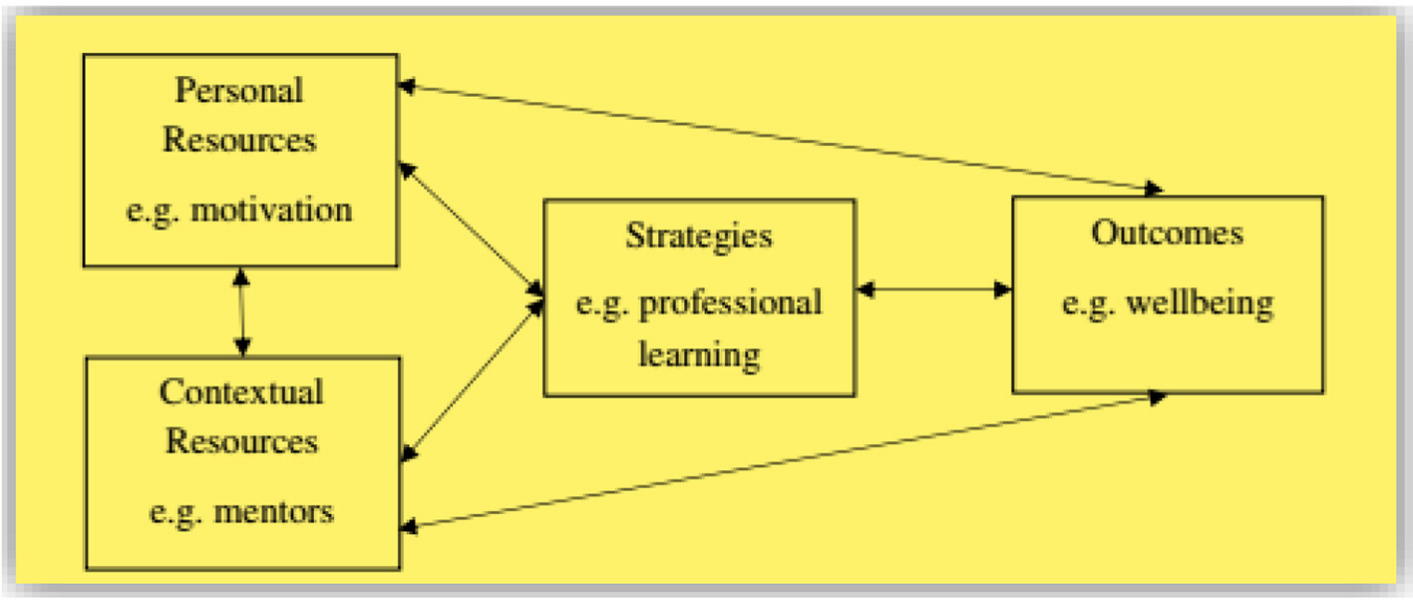
A comprehensive model of building teacher resilience.

The most practical model of implementing resilience in teacher education is that of Mansfield et al. (2016) who regarded resilience as a collective construct growing out of multi-layer systems and ecosystems. Based on this model, resilience is made up of *personal resources, contextual resources, strategies*, and *outcomes* which dynamically interact with each other (Figure 1).

According to this model, teacher resilience is a dynamic process in which different components have to work interactively and collectively to adapt to adversities that challenge a system (i.e., person, community, institution, and ecosystem). Later, the model changed into five modules of *Building Resilience, Relationships, Well-being, Taking Initiative*, and *Emotions* with related topics to be covered in online and ordinary training courses. The model has been used in different educational contexts (USA, Spain, Australia, and The Netherlands) and the results were astonishing in that teachers' resiliency level improved exponentially. That is because in this model the responsibility of constructing teacher resilience is not placed upon only one group's shoulder but all the educational system parties and their relationships. Not being an exception, the teacher education of China can employ this systematic view of teacher resilience to cultivate their teachers' toughness, immunity, and buoyancy when encountering academic challenges. This model fits the socio-cultural characteristics of China in which the aim of teaching and learning is personal and societal development, there is openness and diversity in academia, and education is an integration of knowledge and social action. These features reflect person-focused, process-focused, context-focused, and system-focused dimensions of Mansfield et al.'s 2016 model. Although the model is yet conceptual for Chinese education, it can be easily implemented with a focus on the dynamic, interactive, and systemic nature of teaching and teacher education. However, it may not work in static and teacher-centered classes where there is no interaction among the nested systems of education.

## Practical Applications For Building Teacher Resilience

**Figure 2:**
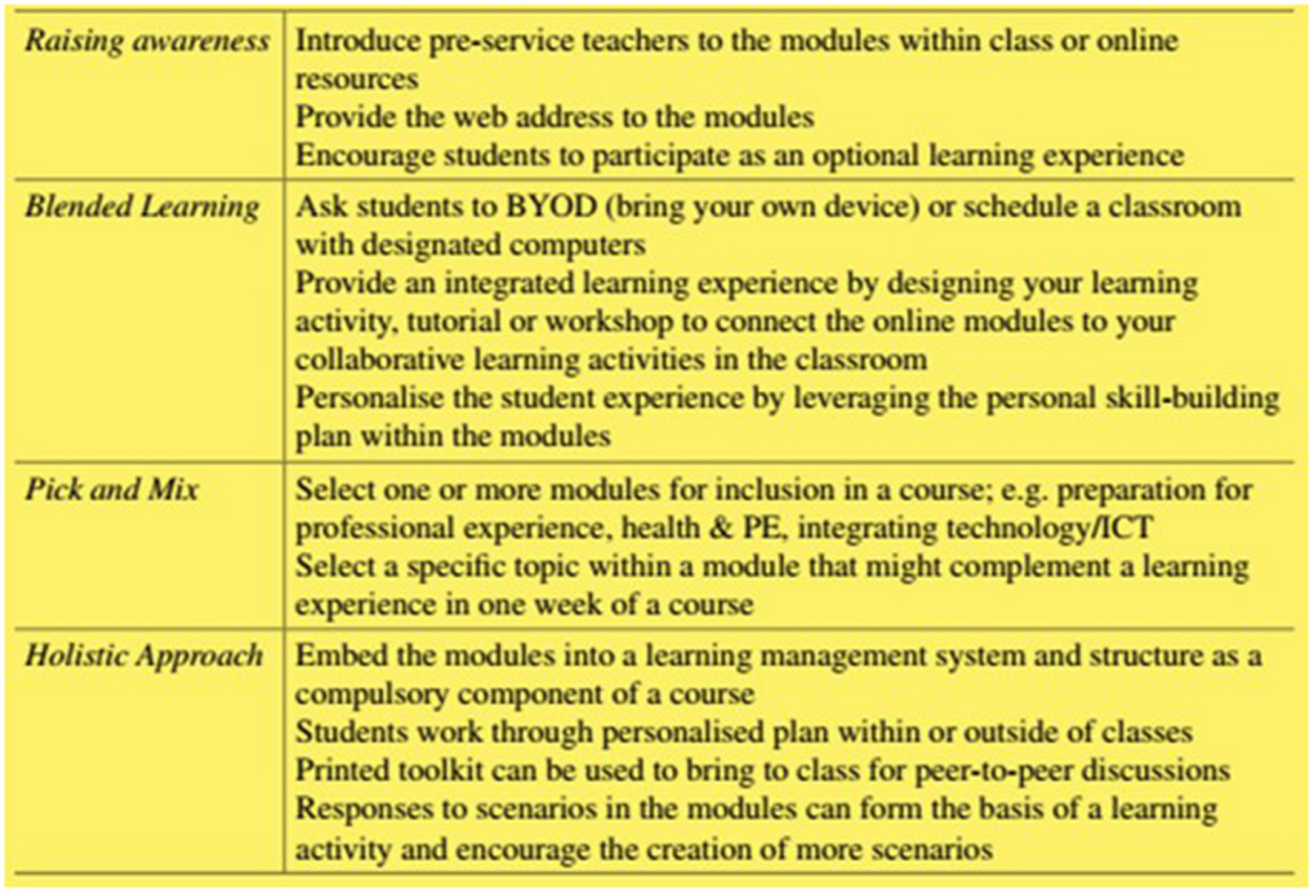
A guide for implementing teacher resilience model in teacher education.

The implementation of Mansfield et al.'s (2016) model in Chinese teacher education has some practical applications. For instance, teacher trainers and program designers can develop online and face-to-face workshops, seminars, webinars, and conferences in which the five modules of the model are comprehensively covered via appropriate tasks and activities whose cumulative outcome would be an augmented teacher resilience. As a universal guide, Mansfield et al. (2016) proposed a sample chart for implementing their model as following (Figure 2):

In addition to building resilience in teachers, this model can be used to assess Chinese teachers' mindfulness, well-being, self-care, positive emotions, and their ability to create a positive educational context that highlights pedagogy, learning, and their emotional aspects.

## Implications and Directions For Future Research

In light of this study, which explored the possibilities of building teacher resiliency in the teacher education of China, it can be concluded that teacher resilience is not an innate characteristic but an improvable trait through training and intervention. To do so, a dynamic and systematic model is needed to cause revolutionary changes in this domain. Drawing on Mansfield et al.'s (2016) seminal framework to implement resilience in teacher education, this article explicated the developmental trajectory of China's teacher education and teacher resilience's conceptualizations, definitions, and significance. Hence, it has precious implications for Chinese teachers, students, teacher education, policymakers, materials developers, and researchers. Teachers can use the results to heighten their awareness and ability to tackle teaching difficulties and setbacks. Likewise, students can help their teachers by establishing a positive relationship in the class which fosters resilience building in teachers. The results are beneficial for teacher education in China in that they can revise their professional development programs by offering effective workshops and seminars in which different modules of teacher resilience are taught along with pedagogical techniques required of a teacher.

Moreover, this study raises the awareness and knowledge of policy-makers to separate education from politics and economy and offer professional development courses oriented toward the instructional and emotional advancement of Chinese teachers at all levels. Likewise, the results would be of help for materials developers in that they can design tasks, activities, and textbooks in which the emotional state of the teacher is considered in a way that he/she can handle adversities with ease. Finally, researchers can benefit from this study in that they can run replication studies using the same model in their own contexts. They can also conduct multidisciplinary studies from different perspectives using suitable instruments that can capture the complexity of teacher resilience. More research is required on the cognates of resilience like buoyancy, coping, and hardiness. Additionally, cross-cultural studies can be done on different components of teacher resilience to see if they are universal or culture-specific. Finally, avid scholars can use qualitative research tools in longitudinal studies to examine the dynamism of teachers' resilience level through time.

## Author Contributions

The author confirms being the sole contributor of this work and has approved it for publication.

## Conflict of Interest

The author declares that the research was conducted in the absence of any commercial or financial relationships that could be construed as a potential conflict of interest.

## Publisher's Note

All claims expressed in this article are solely those of the authors and do not necessarily represent those of their affiliated organizations, or those of the publisher, the editors and the reviewers. Any product that may be evaluated in this article, or claim that may be made by its manufacturer, is not guaranteed or endorsed by the publisher.
